# Looking for Commensality: On Culture, Health, Heritage, and the Mediterranean Diet

**DOI:** 10.3390/ijerph18052605

**Published:** 2021-03-05

**Authors:** Francesc-Xavier Medina

**Affiliations:** Unesco Chair on Food, Culture and Development/FoodLab, Faculty of Health Sciences, Universitat Oberta de Catalunya (UOC), Rambla del Poblenou, 156, 08018 Barcelona, Spain; fxmedina@uoc.edu

**Keywords:** commensality, Mediterranean diet, health, culture, heritage, sustainability, conviviality, eating together

## Abstract

The concept of the Mediterranean Diet has substantially evolved in the last decade and a half. From a model focused uniquely on nutrition and public health, in recent years, and after its registration as Intangible Heritage of the Humanity by the United Nations Educational, Scientific and Cultural Organization (UNESCO), its conception incorporated important elements related to society, culture, and sustainability. In this regard, the use of concepts such as commensality (or conviviality around food, or eating together), linked to a more cultural vision of food, began to be one object of attention. The aim of this article is to reflect on the role of these “new” elements regarding the actual definitions of the Mediterranean diet and, particularly, its relationship with other significant discourses inside this concept, as the preponderant of health, or the emergence of sustainability.

## 1. Introduction

The notion of the Mediterranean diet has undergone a progressive evolution since its creation and over the past 50 years, from that of a healthy dietary pattern (mainly for the heart) to the model of a sustainable diet [1], passing through culture and heritage on the way [2]. This evolution has transformed the concept of the Mediterranean diet from strictly medical and nutritional positions to visions more closely linked to society, culture, and lifestyles [3]. However, and despite the importance of this transformation, the centrality of the perspective related to health continues to be the main axis around which all other aspects rotate.

Nevertheless, being acknowledged in 2010 by the United Nations Educational, Scientific, and Cultural Organization (UNESCO) as an intangible cultural heritage of Humanity, the Mediterranean Diet added different values to its definition. In this regard, and far away from “healthy” arguments, the Mediterranean Diet was inscribed in the UNESCO’s Representative List as a set of skills, knowledge, rituals, symbols, and traditions, ranging from the landscape to the table. This inscription particularly remarks that the fact of eating together is the foundation of the cultural identity and continuity of communities throughout the Mediterranean basin. In this regard, the Mediterranean diet emphasizes values of hospitality, neighborliness, an intercultural dialogue, creativity, and a way of life guided by respect for diversity [4].

The inscribed Mediterranean diet embodies landscapes, natural resources, biodiversity, and associated occupations as well as the fields of health, welfare, creativity, intercultural dialogue, and, at the same time, social and cultural functions and values such as hospitality, commensality, or conviviality [1]. In this regard and from this moment on, the use of concepts such as conviviality or commensality (or eating together, as the declaration itself remarks), linked to a more cultural vision of food, began to be one more relevant object of attention to take part of the future definitions of the Mediterranean diet.

The aim of this article is to approach the use and development of these concepts regarding the Mediterranean diet and to observe to what extent they come to transform its very definition or, on the contrary, they are used from other more powerful discourses (such as health), to enhance their own definitions and interests.

## 2. Food, Commensality, and Eating Together

According to the Merriam Webster Dictionary, the word commensality means: “The practice of eating together” or “a social group that eats together” [5]. This practice seems to be universal, present likely in all cultures, in different places, and periods of history. In an extended sense, and as French sociologist Claude Fischler points out, commensality conveys the notion of sharing food habitually, possibly with an assumption of dependence of one or several of the commensal parties upon another, thus, bringing commensals closer [6]. French sociologist Michel Maffesoli adds that we rarely eat alone. In this regard, eating together has always been one of the foundations of any community [7].

Following Sobal (2000), we can find three different dimensions in sociability: (a) commensality, which means eating together with other people according to societal rules, (b) interaction, which deals with social relationships during meals, and (c) facilitation, which refers to the importance of the social environment on food behavior [8]. In this regard, and following Grignon, commensality has to be distinguished from conviviality [9]. Commensality aims at preserving the social structure of a group. In this way, conviviality would, thus, be only a possible configuration of commensality [10].

Commensality means more than sharing food and sharing time eating together. As W. Roberston Smith wrote more than a century ago, the very act of eating and drinking with someone is a symbol and a confirmation of fellowship and mutual social obligations [11]. In this regard, eating together is usually strongly defined by rigid societal rules. People do not share food (and time) with just anyone. Sharing food is strongly patterned in different cultures based on important aspects, such as gender [2], age [12], class, or social stratification [13]. In some cases, people can share food if there is no other option, but they do not sit together around a table because the affinities that could sit them together to eat are rejected. The willingness to commensality requires the acceptance of affinities that make the table and food symbols of brotherhood or acceptance. Thus, sometimes, we use commensality to manage the difference, not always in a positive way, and commensality with other groups should be problematic [13].

In this regard, and as a starting point, we can establish that commensality is universal, and does not correspond to any specific geographical area or historical moment. It is voluntary, but it is also strongly, socially, and culturally controlled. It brings commensals closer together, but always within identifiable social rules, and, on specific occasions, it serves more to socially divide and establish borders than to unite [13].

## 3. Commensality in the Mediterranean Area: Towards the Establishment of the Basics of the Mediterranean Diet

Commensality is not specifically Mediterranean. However, in some of the cultures that developed around the Mediterranean, it acquired a degree of institutionalization and political meaning that contributed to essential further developments [6]. As Phull states [2], one of the most prominent cultural ideals promoted in the Mediterranean Diet model is the idea of conviviality and the pleasure of shared meals. This author remarks that it is this notion of pleasure that really sets the Mediterranean diet apart from other dietary cultures, defining this diet as ‘palatable’ and not only ‘convenient.’ Following this author, we define that palatability and pleasure are aspects that works in favor of commensality.

In this regard, commensality is considered to be on the bases of the Mediterranean diet [14]. The idea of sharing food around a table and eating together has been considered by several authors as one of the foundational social facts of what today is known as the Mediterranean diet [2,6,9,15].

Looking forward to the origins of the Mediterranean Diet, Berry et al. compare current trends of eating and trends in ancient times. The authors state that current trends of eating while watching television promote unhealthy, quick meals and exclude social/family communication. In biblical times, nevertheless, mealtimes nurtured relationships and was an opportunity for communication. They state that the social setting for eating promotes fixed mealtimes and may overcome the reluctance to change in lifestyles experienced today. The Last Supper was the traditional Passover Seder meal and was styled on the Greek symposium in which people reclined and communicated while eating [16].

It is necessary to note that neither author appears to be a historian or archaeologist (they are all medical researchers), nor is this quote based on any data or supporting literature. This quote, however, is interesting because of what it shows about the relevant role of commensality within the discourse on the origins. Another example can be found in another short medical quote regarding alcohol consumption, taking part of a whole definition of the Mediterranean diet: “Alcohol consumption was common in the traditional Mediterranean diet, but generally in moderation and in the form of wine and, as a rule, during meals in the spirit of the ancient Greek word symposium” [17].

Looking forward, the origins of the Mediterranean diet in biblical texts or in past foundational societies (like the ancient Greece or Rome) serves, on the one hand, to give historical depth and permanence to the discourse, and, on the other hand (and although the first cited article is dedicated solely to the nutritional analysis of seven foods considered to be present in the Bible, and the second one is devoted to highlight the multiple benefits of the Mediterranean Diet regarding health), the presence of social aspects, even if anecdotal, give a more holistic perspective to the idea exposed. Not in vain, the traditional Mediterranean diet (MD) is considered to be the heritage of millennia of exchanges of people, cultures, and foods of all countries around the Mediterranean basin [18].

However, the reference to the Greek symposium is of special importance to our analysis. As British classicist Peter Garnsey points out, the Greek symposium is the same institution that the Roman convivium (from Latin *con-vivivere*, living and staying together): “This was a formal meal at a set time (and the convivium is) the obvious place for interaction, conversation, and relaxation, the place and the occasion where friendship was strengthened and cultural attainment displayed. This picture is confirmed by the importance of the dining-room (or the dinning-rooms), triclinium, in the aristocratic house” [19]. Therefore, the symposium or convivium place the importance of conviviality around food (or commensality) since ancient times. In addition, this commensality seems to be passed down through the centuries (perhaps in a somewhat abrupt way, with little historical basis and without taking into account essential aspects, such as gender or social class, particularly relevant here, if one talks about aristocratic habits), up to the present moment, talking about the Mediterranean Diet.

Nevertheless, and refocusing our analysis, commensality seems to be (still being) in the core of the Mediterranean food traditions. In this regard, the Mediterranean diet and the practices attached to it, derive from multiple gastronomic traditions, originating in different localities and different class strata, which is mixed with time as a result of people’s contacts. As Scarpellini points out, the Mediterranean encompassed lifestyle, a link to locality and region, concern for one’s health, care in preparing food, and places and ways to express conviviality [20].

## 4. From Health to Culture

The Mediterranean Diet is internationally known due to its promotion as a diet beneficial to health. As Dernini et al. pointed out, since the pioneer Seven Countries study, much scientific evidence has highlighted the protective effect of the Mediterranean diet on cardiovascular disease and its health benefits to prevent chronic and degenerative diseases. The beneficial effects of the Mediterranean dietary pattern (MDP) in relation to cardiovascular disease and mortality have long been recognized [1].

In this regard, the concept of the Mediterranean diet came into being shortly after the mid-twentieth century as a recommended tasty and healthy diet [21] mainly aimed at the North American society [22,23]. Since then, it has undergone various modifications that have led it from being a concept linked solely to health, to an element of culture and a lifestyle, as a result of its declaration as an intangible cultural heritage by UNESCO in 2010 by four countries: Greece, Italy, Morocco, and Spain [3] and, again in 2013, with the extension of the candidacy to three new countries: Croatia, Cyprus, and Portugal [3].

This long journey from health to culture especially affected the very conception of the Mediterranean diet, which, from its recognition as an intangible cultural heritage by UNESCO, focuses much more on social and cultural aspects. It is, at this time, that the role of commensality and “eating together” takes on special prominence.

In this regard, the final inscription of the Mediterranean Diet to UNESCO says, specifically, that the Mediterranean Diet ensures important social and cultural functions, and, first of all, bringing people together and strengthening social links. It also states that consumption, which, in the Mediterranean area, means eating together. It is the relational foundation and the guarantee of the identity and of the cultural and social continuity of the communities and their individual [4].

This final inscription also stated that eating together in the identified Mediterranean communities is a moment of intensity and solemnity, more or less explicit, but always felt of social exchange and of communication. In addition, they quote the classics to say that it is a millenary inherited aspect of daily life: ‘We do not sit at table to eat but to eat together’ (Plutarch). In addition, they categorically affirm that, today, this aspect remains untouched.

It is interesting to read that meeting around a table for a meal is a ritual, an ‘almost religious’ moment of affirmation and of ‘rebuilding’ of the family, the group, or the community, including its values, history, environment, symbols, beliefs, and its way of life. It is an occasion to share the present and to establish the future. On occasion of religious or secular festivities or rites of passage, these features are particularly broadened. This text also added that words have a major place at the table to tell, transmit, appreciate, present, and celebrate. The ritual conversations at the end of a meal (“sobremesas” or “terdida”) are decisive contributions [4].

Beyond the informative and non-academic language of an official document such as that of this inscription, the fact of the commensality appearance (eating together) in such a prominent place among the cultural aspects related to the Mediterranean diet (in the first position of all of them) indicates, to a large extent, the importance of this concept within the proposal. This quoted grey text recovers some of the aspects that I already discussed previously: a millenary inherited aspect of daily life that today remain untouched, the relational foundation and the guarantee of the identity and continuity of the communities, and a moment of affirmation and of rebuilding the group. This inscription adds an occasion to share the present and to establish the future.

However, the importance of this text and, in general, of the definitive inscription of this heritage by UNESCO in 2010 and its extension in 2013, goes beyond the declaration itself. Some of its direct consequences matter particularly. First of all, and as I already noted before, 2013 saw the adherence to the UNESCO inscription of the Mediterranean diet of three new countries: Croatia, Cyprus, and Portugal. Since 2013, there have been seven countries that consider this heritage as their own in an official way. In this regard, it is also important to remark that this declaration remain open, so further affiliation of other Mediterranean countries is still possible in the future.

However, the most remarkable consequence of this declaration is the reformulation of the pyramid of the Mediterranean Diet. One year after the inscription by UNESCO, one article titled *Mediterranean Diet Pyramid Today: Science and Cultural Updates* was published by an interdisciplinary group of researchers (some of them are related to the candidature). In this article, the prominence of cultural aspects in relation to the Mediterranean Diet, and, although it continues to have a subordinate role regarding its health benefits, acquires a more significant role.

In the justification of the article, the authors say that any innovations have arisen since previous graphical representations of the MD. First, the concept of composition of the ‘main meals’ is introduced to reinforce the plant-based core of the dietary pattern. Second, frugality and moderation is emphasized because of the major public health challenge of obesity. Third, qualitative cultural and lifestyle elements are taken into account, such as conviviality, culinary activities, physical activity, and adequate rest, along with proportion and frequency recommendations of food consumption. These innovations are made without omitting other items associated with the production, selection, processing, and consumption of foods, such as seasonality, biodiversity, and traditional, local, and eco-friendly products [18].

This statement of intent is important. On the one hand, the statement of intent is for granting a relevant role (although, as we see, placed in the third and last place of the general arguments) to food culture among the contributions of the article. Additionally, on the other hand, in relation to the topic at hand, to mention the conviviality related to food (that is, to a large extent, commensality) is the first aspect to highlight among all the cultural aspects mentioned. In this regard, this article includes a specific item on “socialization” following the guidelines of the declaration by UNESCO: “Socialization: The aspect of conviviality is important for the social and cultural value of the meal beyond nutritional aspects. Cooking, sitting around the table, and sharing food in the company of family and friends is a social support and gives a sense of community” [18].

Then, in the same paragraph, the authors add: “In this sense, several factors related to food (understood as a social fact) must be kept in mind, such as culinary activities, knowledge transmitted from generation to generation, and time devoted to meals related to the daily pace of life…. The pleasure associated with the conviviality of meals may positively affect food behaviors, and, in return, health status” [18].

This last sentence leads us to a new element of analysis that is interesting to our eyes: the nexus between culture, nutrition, and health. In this way, conviviality-commensality may also positively affect the health status. This is an important aspect that links to health promotion (which we cannot forget is still the main objective of this article). It is not only important what you eat, but how you eat it, with whom you eat, in what situation, and for how long.

Following this argument, Fischler points out that, in the United States and, to a certain extent, in Britain, eating has become increasingly individualized and medicalized. It is considered a form of private consumption. In Italy or France, in contrast, it is more structured around mealtimes and commensality with an essential social (public) dimension [24]. This author also states that one can observe some of those nations show attachment to commensality, generally seem to fare better in terms of obesity and related health problems [24]. Even if other authors consider that the relationship between commensality and health is ambivalent (particularly regarding a negative influence with regard to overconsumption of alcoholic beverages or fat food), we can also say that it has a positive impact in general terms, particularly in healthy people [9,25].

Both perspectives seem to converge on the fact that, beyond ingested nutrients or energy, commensality seems to play an important role, not only regarding culture, but also health. Thus, after this perspective, we have found that commensality should be considered an essential concept and object of research in nutrition [24].

## 5. Mediterranean Diet, Sustainability, and the Eventual Role Played by Commensality

In 2011, the International Centre for Advanced Mediterranean Agronomic Studies (CIHEAM) and the Food and Agriculture Organization of the United Nations (FAO) identified the Mediterranean diet as a case study for characterization and assessment of the sustainability of dietary patterns. After different expert meetings under the auspices of the two supranational organizations, the Mediterranean diet was going to be observed as a ‘sustainable food system’ in the Mediterranean area. In 2017, an article was published, which can also be considered as programmatic for this new stage: *Med Diet 4.0: The Mediterranean Diet with Four Sustainable Benefits* [1].

The new framework in which this article is based arises from the previous analysis of the definitions of sustainable diets, sponsored by the Food and Agriculture Agency of the United Nations (FAO) [26], and includes the following points: (1) health: healthy and nutritionally adequate, (2) sustainability: low environmental impact, respectful, and protective of biodiversity and ecosystem, (3) culture: culturally acceptable, and (4) economy: accessible and affordable. Within the three basic pillars: social, environmental, and economic, this quoted article by Dernini et al. added sustainability as a cross-cutting element as well as a primary objective.

As Dernini et al. points out, the Mediterranean Diet 4.0 methodological approach tries to contribute to understanding what constitutes locally a sustainable diet in the different agri-ecological Mediterranean zones, by taking into account the regional variations and country specificities within the Mediterranean region. Having this in mind, four thematic sustainability dimensions of the Mediterranean Diet were also identified: health and nutrition, biodiversity and environment, social and cultural factors, and economic aspects. As an outcome of this interdisciplinary and multidimensional sustainability approach, four benefits were highlighted in one single comprehensive Med Diet 4.0 framework, with country-specific variations: major health and nutrition outcomes, low environmental impacts and richness in biodiversity, high sociocultural food values, and a positive economic return locally [1].

In any case, what I am most interested in observing here is the specific role of the cultural item, and, within it, that of commensality as a subject of analysis. In this regard (and even if briefly), the links between cultural aspects and food sustainability are pointed out and, among them, the importance of conviviality around food. Thus, and as this article [1] points out, in the Mediterranean cultures, eating is important over and above the physiological need for energy. Following this premise, it states that family and communal meals are a moment of conviviality and importance, as well as fun and pleasure (more or less explicit) and represent a daily opportunity for social exchange and communication.

In this regard, we can observe that, beyond nutrition and, also in this case, beyond the social and cultural aspects themselves, commensality (or more specifically, conviviality related to food, as specified in this case) is an aspect that plays in favor of sustainability, in the same way that we had previously seen that it was “an essential concept and object of research in nutrition.” An interesting aspect to note is that some authors, such as Truzzi et al., include commensality as an aspect linked to sustainability, among others. Thus, they say that the Mediterranean Diet promotes social interaction. It has given rise to a considerable body of cultural habits, it is deeply rooted in a local territory, it protects biodiversity, and it ensures the conservation and development of traditional activities. They also add that the next target will be to adapt the Mediterranean Model to local food and traditions [27].

## 6. Conclusions: Commensality as a Cultural Driver Related to the Mediterranean Diet

Throughout this article I tried to review some aspects related to the use of the concept of “commensality” (or conviviality around food, eating together) regarding the Mediterranean diet as an object of study. In this regard, we observed, on the one hand, the evolution that the concept of the Mediterranean diet has undergone in the last decades and, particularly, after 2010 (year of the inscription of the Mediterranean diet as Intangible Cultural Heritage of the Humanity by UNESCO) from strictly nutritional-medical positions to visions more closely linked with society, culture, and lifestyles and, beyond this, to the sustainability in the Mediterranean area. On the other hand, we observed the use of the concept of commensality especially after this moment, when it began to be the object of attention, even to be considered as a substantial part of the future definitions of the Mediterranean diet.

Regarding the first point, there is no doubt that, especially in the last decade, the concept of the Mediterranean diet (created in the 1960s) began a slow but revolutionary evolution, which led it to include elements of a social, cultural, and even environmental nature in relation to its definition itself. Those elements have changed the traditional conception of the Mediterranean diet. Regarding a healthy dietary pattern prescribed by doctors and nutritionists, the Mediterranean diet has come to be considered a cultural and heritage element that includes the lifestyles of the societies and cultures that inhabit the Mediterranean and, more recently, it begun to be observed as an eventual driving force behind responsible, local, and sustainable consumption, as a model of a sustainable development [1].

On the second of the points, and within this panorama, the role of commensality seems to have found a privileged place to define the importance of cultural aspects related to the Mediterranean diet. Even if this aspect cannot be specifically considered as Mediterranean, it is considered to play a particular role in likely the totality of the cultures that developed around the Mediterranean region and, following Fischler [24] talking about ancient historical-cultural aspects like the sacrificial banquet, the Jewish Shabbat meal or the Cristian commemoration of the Last Supper in the Eucharist, commensality even acquired a degree of institutionalization and political meaning that contributed to essential, further developments.

Following this, we observed that commensality should have a role in health promotion as an interesting concept and object of research in nutrition [8,9]. Interdisciplinarity should play an essential role [28], and cultural and nutritional aspects are fundamental parts of this comprehensive Mediterranean model or lifestyle [29]. This statement is not new and corresponds to an old claim on the part of food anthropologists [30] 3/5/2021 4:37:00 PMand other social scientists. They argued that preventive medicine would benefit from a more holistic approach to nutritional factors and conditions. There is a very important contribution to health sciences in the holistic approach to food and nutrition, where social and cultural perspectives are included with nutritional biology in the study of epidemiological diversity and medical needs [31,32].

However, while it can be noted that some aspects have evolved significantly in recent years, the Mediterranean diet carries a significant bias related to health, which will be difficult to get rid of. Even if we can collectively agree on the fact that the Mediterranean diet undergoes a conceptual transformation over recent years, which has taken it from unique health promotion to include sustainability. By way of culture, it is also evident that the focus on health has never really declined in importance, nor in prominence. On the contrary, as we have seen through the articles analyzed, the health perspective remains its primary focus.

The Mediterranean diet is a concept that embraces biodiversity, sustainability, quality, palatability, health, and cultural aspects and heritage. Safeguarding the Mediterranean diet as a system should be the driving force behind responsible, local, and sustainable consumption, as a model of a sustainable development. From this perspective, these elements (including commensality), which have been added from interdisciplinary perspectives to the very definition of the Mediterranean diet, they still need a better fit in current definitions and, by the moment, do not cease to have a merely testimonial value. Thus, they are used (are still being used) from other more powerful discourses (mainly health) to enhance their own definitions and interests. The inclusion of interdisciplinary perspectives and their interpretation from open and uninterested perspectives should enrich definitions and allow future collaborations between professionals from different disciplines.

## Data Availability

Not applicable.
